# High Prevalence of Cold Intolerance in Rheumatoid Arthritis: A Case–Control Study

**DOI:** 10.1002/ccr3.71502

**Published:** 2025-11-23

**Authors:** Mohsen Soroush, Rojin Farzaneh, Elaheh Babapour, Amirreza Jabbaripour Sarmadian, Sami Rassouli, Golrokh Hassanzadeh Behrouz, Amirreza Naseri, Maryam Mahmoudi, Kamal Esalatmanesh, Aida Malek Mahdavi, Alireza Khabbazi

**Affiliations:** ^1^ Department of Internal Medicine AJA University of Medical Sciences Tehran Iran; ^2^ Connective Tissue Diseases Research Center Tabriz University of Medical Sciences Tabriz Iran; ^3^ Student Research Committee Tabriz University of Medical Sciences Tabriz Iran; ^4^ Department of Internal Medicine, Faculty of Medicine Kashan University of Medical Sciences Kashan Iran; ^5^ Tuberculosis and Lung Disease Research Center Tabriz University of Medical Sciences Tabriz Iran

**Keywords:** cold intolerance, cold sensitivity, cold‐related symptoms, rheumatoid arthritis

## Abstract

Cold intolerance is more common in rheumatoid arthritis patients than control. Cold intolerance tends to develop after rheumatoid arthritis onset and is associated with longer disease duration, with pain and swelling being dominant features. Cold intolerance leads to a reduced quality of life in rheumatoid arthritis patients.

## 1 Introduction

Cold intolerance includes signs and symptoms such as pain, numbness, stiffness, weakness, and swelling when exposed to cold, sometimes forcing a person to wear gloves or stay indoors [1]. Cold weather is commonly cited as a trigger for musculoskeletal pain [2] and cold intolerance is a common health problem, reported to affect 5%–15% of the general population [3, 4, 5, 6]. Cold intolerance is more common in females, thin people and people with a family history of cold intolerance or those with underlying conditions such as high blood pressure, diabetes, and etc. [5, 6, 7, 8] It should be noted that, cold intolerance, characterized by altered sensory and pain responses to cold without objective vascular signs, is distinct from Raynaud's phenomenon, which results from vasospasm of small arteries with visible color changes [9, 10].

Cold intolerance has many causes, the most important of which are upper limb injuries including trauma and frostbite, upper limb surgeries, fibromyalgia (FM), anemia, hypothyroidism, vascular diseases, Raynaud's disease, diabetes, low body weight and rheumatic diseases [6, 11, 12]. A nested case–control study conducted in Norway showed that rheumatic diseases were a risk factor for cold intolerance with an odds ratio (OR) of 3.1 [6]. However, this study does not provide information about the type of rheumatic disease and the association between cold intolerance and the course of rheumatic diseases [6]. Cold intolerance may have a profound effect on quality of life and may lead to profound disability [4].

Rheumatoid arthritis (RA) is the most common rheumatic disease [13]. The hallmark of RA is symmetrical synovitis of multiple joints, especially the small joints of the hands [14]. Nevertheless, involvement of extra musculoskeletal organs including skin, eyes, lungs, heart, kidneys, blood vessels, salivary glands, central and peripheral nervous systems, and bone marrow occurs in more than 40% of RA patients [14, 15]. During its course, RA causes many health problems for the patient, and the emergence of cold intolerance may add to the patient's health problems and lead to more disability. In this study, we investigated the prevalence of cold intolerance in RA patients and its association with the clinical characteristics and activity of the disease.

## 2 Case Descriptions

### 2.1 Case History and Examination

This case control study included 282 consecutive RA patients regularly followed in the RA clinic of Tabriz University of Medical Sciences (TUOMS). Inclusion criteria for the RA group were (i) fulfilling the American College of Rheumatology/European League Against Rheumatism (ACR/EULAR) 2010 criteria for RA, (ii) age ≥ 18. Patients with overlapping autoimmune diseases were excluded from the study. Four hundred nineteen people with age ≥ 18 were included in the study as a control group from the normal population covered by Emamieh health center in the west of Tabriz city. Tabriz has a continental climate with regular seasons and borders on a cold semi‐arid climate, and the study was conducted in autumn and winter, i.e., from October 23, 2023 to February 19, 2024.

### 2.2 Differential Diagnosis, Investigations, and Treatment

An interview with RA patients was performed during regular visits by a trained researcher. A telephone interview was conducted with the controls by the same researcher. According to the closed follow‐up of RA patients and the normal population covered by Emamieh health center, the participation rates of the RA and control groups were 95% and 90%, respectively. To minimize potential bias between face‐to‐face interviews with RA patients and telephone interviews with controls, all interviews were conducted by the same trained researcher using a standardized questionnaire. The structured format and detailed instructions were designed to reduce interviewer‐related variability. A questionnaire contained questions about demographic characteristics, medications, comorbidities, history of frostbite, history of hand injuries and surgeries, cold‐related symptoms, family history of cold‐related symptoms, duration of cold intolerance and the effect of cold intolerance on job in both groups and disease duration, Disease Activity Score 28 (DAS‐28), rheumatoid factor, anti‐citrullinated peptide antibody in the RA group was filled out during the interview and rechecked by their medical records. Cold‐related symptoms were defined as the presence of one of the following symptoms: pain, numbness, shivering, skin discoloration, weakness, stiffness, or swelling when exposed to cold. In participants with cold‐related symptoms, a Cold Intolerance Symptom Severity (CISS) questionnaire was filled out by the interviewer. This questionnaire reports the intensity of cold intolerance in the form of scores between 4 and 100, and higher scores indicate greater intensity of sensitivity to cold [16]. A CISS score ≥ 50 was defined as cold intolerance [17]. CISS ≥ 50 is used as the threshold because it identifies patients with moderate‐to‐severe cold intolerance that is clinically relevant, based on validation studies in peripheral nerve and musculoskeletal disorders [17]. We used the Farsi version of CISS which was validated previously [5]. Due to the presence of a significant number of illiterate and low‐literate people among the participants, the CISS questionnaire was completed by the researcher during the interview.

Outcomes of RA were evaluated by disease activity, long‐term remission rate and occurrence of irreversible articular damage. Disease activity was assessed by Disease Activity Score‐28 with erythrocyte sedimentation rate (DAS28‐ESR). Long‐term remission was defined by meeting American Rheumatism Association (ARA) criteria for remission [18] and prednisolone dose ≤ 5 mg/d for at least 5 years [19]. Disease‐modifying antirheumatic drugs were permitted. Irreversible articular damage was assessed clinically and defined as limitation of motion or deformity related to RA.

We used the SPSS version 22 (SPSS Inc., USA) for statistical analyses. The normal distribution of data was assessed by the Kolmogorov–Smirnov test. Categorical data were presented as numbers (percentages). Continuous variables with normal distribution were presented as means ± standard deviation and with non‐normal distribution as the median interquartile range (25%–75%). RA and control groups were compared by the Chi‐squared test and Independent‐sample t‐test. To reduce the heterogeneity between RA and control groups, propensity score matching (PSM) analyses were performed. We used 4 parameters including age, sex, body mass index (BMI) and educational status for matching. For each participant with RA, one healthy control was selected (ratio 1:1). Then a multivariate analysis was performed with cold intolerance as the main outcome variable, RA as the main predictor variable and age, sex, educational level, and BMI as covariates. A P‐value less than 0.05 was considered significant.

### 2.3 Outcome and Follow‐Up

**FIGURE 1:**
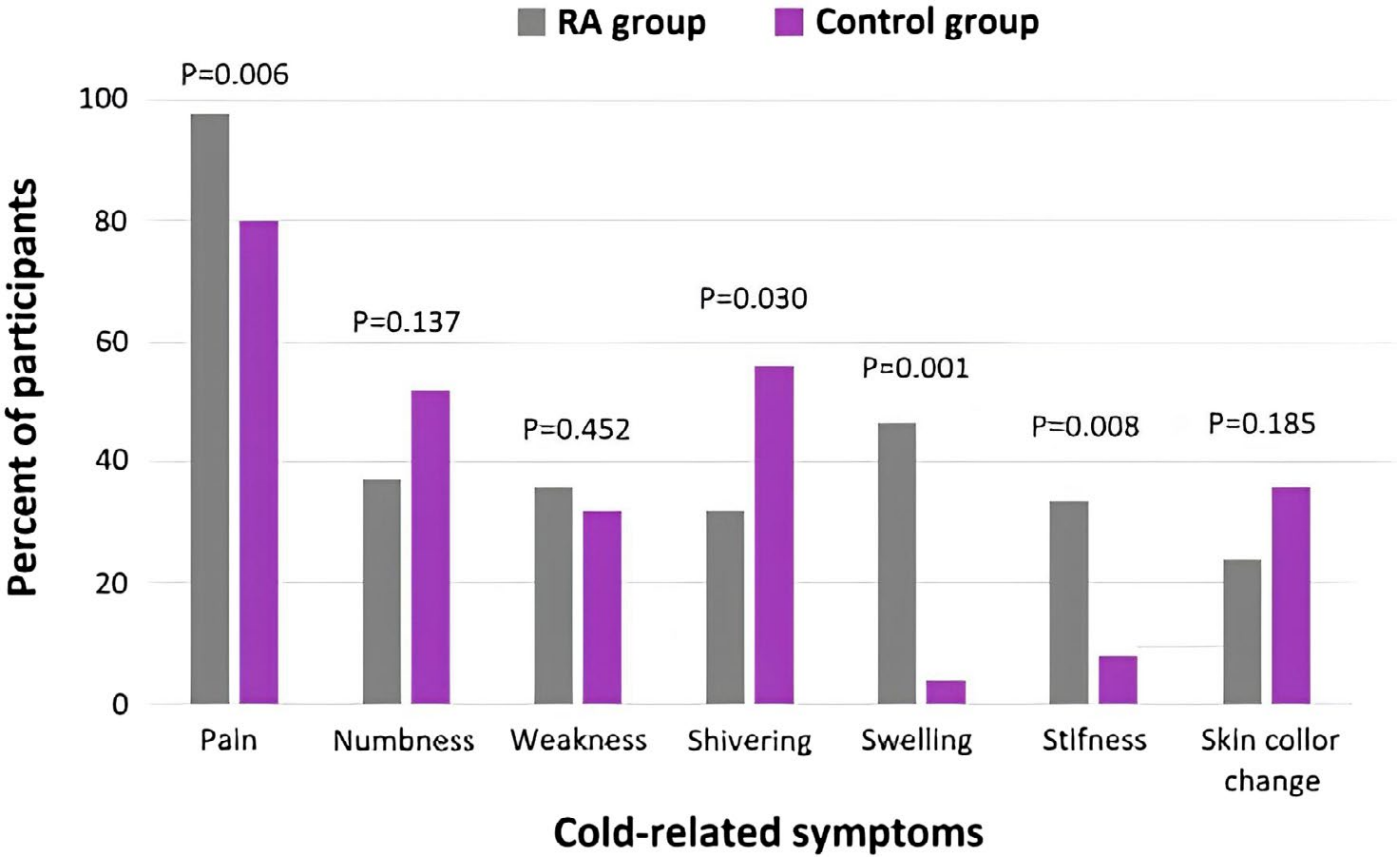
Prevalence of cold‐related symptoms in participants with cold intolerance.

A total of 282 patients with RA and 419 healthy controls were enrolled in the study. PSM resulted in 213 RA patients and 213 matched controls. The demographic and clinical characteristics of participants were shown in Table 1. Cold‐related symptoms were reported in 172 (80.8%) RA patients and 74 (34.7%) healthy controls (Table 2). Cold intolerance according to CISS ≥ 50 was reported in 84 (40.4%) RA patients and 25 (11.7%) healthy controls (Table 2). There was a significant increase in the risk of cold intolerance in multivariate analysis after adjustment for age, sex, BMI and educational level (Table 3). Estimated OR was 6.02 (95% CI 3.52–10.31). Mean age of RA patients at the time of starting cold intolerance was 35.9 ± 16.4 (Table 2). Cold intolerance was started in 72 (83.7%) RA patients after starting the disease. There was no significant difference in the age of starting cold intolerance between RA and control groups (Table 2). Pain was the most common cold‐related symptom in the studied groups (Table 2). Comparison of cold‐related symptoms in patients with cold intolerance, revealed that the prevalence of pain and swelling in RA patients is significantly more than in the control group (Figure 1). Shivering was more prevalent in the control group than in the RA group (Figure 1).

**TABLE 1:** Demographic characteristics of participants after PSM.

Variables	RA group (*n* = 213)	Control group (*n* = 213)	*p*
Age (mean ± SD), years	50.6 ± 12.6	50.4 ± 12.3	0.902
Female (%)	148 (69.5)	142 (66.7)	0.302
Education			
Illiterate (%)	36 (16.9)	37 (17.4)	0.458
Primary school (%)	82 (38.5)	79 (37.1)	
High school (%)	58 (27.2)	59 (27.7)	
University (%)	37 (17.4)	38 (17.8)	
BMI (kg/m^2^)	27.5 ± 4.9	27.8 ± 4.2	0.48
Smoking (%)	21 (9.9)	18 (8.5)	0.416
Disease duration, median (IQR) months	84 (47, 156)	—	
Autoantibodies			
Positive RF (%)	144 (67.6)		
Positive ACPA (%)	161 (75.6)		
Medications			
Prednisolone (%)	110 (51.6)		
Hydroxychloroquine (%)	161 (75.6)		
Methotrexate (%)	153 (71.8)		
Sulfasalazine (%)	18 (8.5)		
Leflunomide (%)	47 (22.1)		
Biologics (%)	8 (3.8)		

Abbreviations: BMI, body mass index; IQR, interquartile range; PSM, propensity score matched; RA, rheumatoid arthritis; RF, rheumatoid arthritis; SD, standard deviation.

**TABLE 2:** Cold‐related symptoms and cold intolerance in participants.

Parameters	RA group (*N* = 213)	Control group (*N* = 213)	*p*	OR (95% CI)
Cold‐related symptoms (%)	172 (80.8)	74 (34.7)	0.001	7.9 (5.1–12.3)
Pain (%)	164 (77.0)	55 (25.8)	0.001	9.6 (6.2–14.9)
Numbness (%)	47 (22.1)	31 (14.6)	0.030	1.6 (1.0–2.7)
Shivering (%)	48 (22.5)	26 (12.2)	0.003	2.1 (1.2–3.5)
Skin color change (%)	32 (15.0)	15 (7.0)	0.006	2.3 (1.2–4.5)
Weakness (%)	42 (19.5)	14 (6.6)	0.001	3.5 (1.8–6.6)
Stiffness (%)	43 (20.2)	3 (1.4)	0.001	17.7 (5.4–58.1)
Swelling (%)	61 (28.6)	4 (1.9)	0.001	20.9 (7.5–58.9)
Cold intolerance (%)	84 (40.4)	25 (11.7)	0.001	5.1 (3.1–8.4)
Age of starting cold intolerance (mean ± SD)	35.9 ± 16.4	33.1 ± 19.2	0.466	
CISS score (mean ± SD)	49.2 ± 19.6	41.3 ± 20.4	0.005	
Affecting involvement in hobbies and interests (%)	99 (46.5)	18 (8.5)	0.001	9.4 (5.4–16.4)
Changing job because of cold intolerance (%)	8 (3.8)	3 (1.4)	0.110	2.7 (0.7–10.4)

Abbreviations: CI, confidence interval; CISS, Cold Intolerance Symptom Severity OR, odds ratio; RA, rheumatoid arthritis; SD, standard deviation.

**TABLE 3:** Multivariate regression analysis of the association between cold. intolerance and RA after PSM.

Variables	Healthy control group		
	OR	95% CI	*p*
Model: (age, sex, BMI and education)			
No cold intolerance	—	—	—
Cold intolerance	6.02	3.52–10.31	0.001

Abbreviations: BMI, body mass index; CI, 95% confidence interval; OR, Odds Ratio; 95%; PSM, propensity score matching; RA, rheumatoid arthritis.

In addition, we compared the demographic and clinical characteristics of RA patients with and without cold intolerance (Table 4). There was no significant difference in demographic characteristics, smoking status and comorbidities in RA patients with and without cold intolerance (Table 4). However, disease duration was significantly longer in RA patients with cold intolerance (Table 4). Familial history of cold‐related symptoms was present in 31 (36.0%) of RA patients with cold intolerance and 40 (31.5%) of RA patients without cold intolerance (*p* = 0.293). These figures for healthy controls with and without cold intolerance were 12 (48.0%) and 32 (17.0%), respectively. The difference was significant (*p* = 0.001). There was no significant difference in the rate of working in a cold environment between RA patients with and without cold intolerance (Table 4). In addition, the difference in the frequency of hand joint involvement and RA outcomes including long‐term remission and poor joint outcome in the two groups was not significant (Table 4). Cold intolerance significantly affected involvement in hobbies and interests in RA patients (Table 4).

**TABLE 4:** Comparison of demographic and clinical characteristics of RA patients with and without cold intolerance.

Parameters	Cold intolerance (*N* = 86)	No cold intolerance (*N* = 127)	*p*	OR (95% CI)
Age at disease onset (mean ± SD), years	39.7 ± 12.6	42.4 ± 13.4	0.923	
Age at analysis (mean ± SD), years	50.7 ± 12.4	50.5 ± 12.8	0.137	
Female (%)	61 (70.9)	87 (68.5)	0.412	1.1 (0.6–2.0)
Smoking (%)	9 (10.5)	12 (9.4)	0.491	1.1 (0.5–2.8)
Disease duration, median (IQR) months	108 (48,168)	72 (36,144)	0.028	
Comorbidities (%)	53 (61.6)	88 (69.3)	0.156	0.8 (0.4–1.3)
Hypertension (%)	15 (17.4)	29 (22.8)	0.083	0.6 (0.4–1.2)
Diabetes (%)	11 (12.8)	18 (14.2)	0.470	0.9 (0.4–2.0)
Thyroid disorders (%)	2 (2.3)	10 (7.9)	0.078	0.3 (0.1–1.3)
Fibromyalgia (%)	13 (15.1)	22 (17.3)	0.409	0.9 (0.4–1.8)
Peripheral vascular diseases (%)	0	0		
Upper limb trauma, diseases and surgeries (%)	40 (46.5)	42 (33.1)	0.033	1.8 (1.1–3.2)
Raynaud's phenomenon (%)	12 (14.0)	13 (10.2)	0.269	1.5 (0.6–3.4)
Familial history of cold intolerance (%)	31 (36.0)	40 (31.5)	0.293	1.3 (0.7–2.3)
Working in cold place (%)	12 (14.0)	14 (11.0)	0.332	1.4 (0.6–3.1)
Hand joints outcome (%)	83 (96.5)	124 (97.6)	0.506	0.6 (0.1–4.5)
Poor joint outcome (%)	44 (51.2)	65 (51.2)	0.558	1.1 (0.6–2.0)
Long term remission (%)	14 (16.3)	17 (13.4)	0.452	1.2 (0.5–3.2)
Affecting involvement in hobbies and interests (%)	62 (72.1)	37 (29.1)	0.001	6.3 (3.4–11.5)
DAS‐28 at cohort entry (mean ± SD)	4.5 ± 1.1	4.5 ± 1.3	0.917	
DAS‐28 at analysis (mean ± SD)	2.8 ± 0.9	2.5 ± 0.7	0.009	

Abbreviations: CI, 95% confidence interval; DAS‐28, Disease Activity Score‐28; IQR, interquartile range; n, number; OR, Odds Ratio; 95%; RA, rheumatoid arthritis; SD, standard deviation.

## 3 Discussion

Environmental factors have an important role in susceptibility to RA. One environmental factor that has been suspected for centuries to be associated with RA is exposure to cold environments. Musculoskeletal disorders are influenced by cold exposure, which has been associated with increased pain perception and higher prevalence of musculoskeletal complaints in colder regions [20, 21]. Chronic cold exposure induces collagen‐induced arthritis in rats by causing dysbiosis of the gut microbiota [22]. Mechanisms including spinal microglia activation, and elevation of interleukin (IL)‐1β and IL‐6, have been suggested for the development of inflammatory pain in cold weather [23, 24, 25]. The relationship between cold and RA is complex. In a population‐based study, Zeng et al. [26] showed that working in a cold environment is a risk factor for RA (OR = 1.5) and there is a dose‐dependent relationship between working in cold weather and developing RA. Several studies reported that RA disease activity decreases in warm seasons and increases in cold seasons, and the increase in RA activity in cold seasons is not only dependent on the patients' perception of pain in cold weather, but also on the number of swollen joints, which increases significantly [27, 28]. The seasonality of RA activity can be explained by vitamin D deficiency and immune system activity changes in cold seasons, especially increased expression of pro‐inflammatory genes in peripheral blood mononuclear cells [29, 30, 31]. It has been suggested that genetic and epigenetic machinery, especially DNA methylation and non‐coding RNAs, by changing immune response play a role in seasonal flares of RA [32]. The present study investigated another aspect of the association between RA and cold, showing that cold‐related symptoms and cold intolerance are significantly more common in RA patients than in the general population. Most patients reported that cold intolerance began after the onset of RA and was significantly associated with disease duration and the presence of upper limb trauma, diseases and surgeries. In addition, it had no associations with sex, age, family history of cold intolerance, working in a cold place, involvement of hand joints and disease activity.

Non‐noxious temperature is detected in the skin and mucous membrane by cold thermoreceptors [33]. The quality of sensations caused by the cold is very variable, ranging from freshness to unpleasant coldness or even severe pain [33]. Increased sensitivity to cold is a common complication of peripheral nerve damage after limb trauma or surgeries [34]. Although the mechanisms underlying cold hypersensitivity after nerve injury are not understood well, however, altered processing by second‐order spinal cord sensory neurons of the information provided by cold receptors [35] and changes in ion channel expression at peripheral cold‐specific terminals [36] following injury have been suggested. Abnormality in pain processing has been reported in RA patients [37]. Peripheral sensitization (nociceptors sensitization) is one of the explanations for this phenomenon [37]. Inflammatory cytokines, including tumor necrosis factor‐α, IL‐1β, IL‐6 and IL‐17 by activation of intracellular signaling pathways of nociceptor neurons and dorsal root ganglion neurons reduce the threshold to generate action potentials and sensation of pain [37, 38, 39]. However, it should be noted that although with treatment of RA and control of inflammation and developing remission, pain decreases in these patients, pain scores in them are greater than the general population [40]. Peripheral neuropathy especially, entrapment neuropathy may be another explanation for cold intolerance in these patients [41]. In confirmation of this hypothesis, we found the prevalence of upper limb trauma, diseases and surgeries was higher in RA patients with cold intolerance than in RA patients without cold intolerance. Dysregulation of central nervous system (CNS) regulatory mechanisms may contribute to abnormal pain processing in RA [37]. This mechanism has been widely introduced as a cause of pain in FM and osteoarthritis [37, 42]. Wartolowska et al. showed that RA patients have larger volumes of the caudate nucleus, putamen, and nucleus accumbens, which play an important role in the sensory‐differentiation processing of pain [43]. Being DAS‐28 equal at cohort entry and having higher DAS‐28 in RA patients with cold intolerance may refer to this phenomenon that pain sensitization due to central causes, rather than inflammation itself may contribute to developing higher DAS‐28 [44]. However, it should be noted that there was no significant difference in the frequency of FM in RA patients with and without cold intolerance in our study. Finally, cognitive misattribution may be another explanation for the higher frequency of cold intolerance in RA patients. According to the general belief that there is a correlation between rheumatism and cold, the patient may focus more on musculoskeletal pain in exposure to cold and attribute it to the cold [45]. Environmental factors like frostbite and working in a cold environment are a risk factor for developing cold intolerance in the normal population [4], however in RA patients with and without cold intolerance no difference was observed in the rate of working in a cold place.

The strength of this study was the high response rate and the matching of the RA group with the control group by PSM, which reduces the possibility of selection bias and the interaction of potential confounding factors. However, it should be noted that information on the onset date of cold sensitivity is based on patient recall, which can lead to recall bias. The exclusion of potential confounding factors in the association between RA and cold intolerance, such as trauma or upper limb surgery, from the regression analysis, which may be more common in the RA group, is another limitation of the study. Other limitations were single‐center design, and lack of objective vascular or nerve conduction testing to confirm cold intolerance pathophysiology.

Cold intolerance in RA is a common phenomenon and most patients report that cold intolerance began after the onset of the disease Various factors such as inflammation, disturbed pain processing, peripheral nervous system involvement and cognitive misattribution may play a role in its development.

## Author Contributions

**Mohsen Soroush:** data curation, writing – review and editing. **Rojin Farzaneh:** data curation. **Elaheh Babapour:** data curation. **Amirreza Jabbaripour Sarmadian:** writing – review and editing. **Sami Rassouli:** data curation. **Golrokh Hassanzadeh Behrouz:** writing – review and editing. **Amirreza Naseri:** writing – review and editing. **Maryam Mahmoudi:** data curation, writing – review and editing. **Kamal Esalatmanesh:** data curation, writing – review and editing. **Aida Malek Mahdavi:** data curation, project administration, supervision. **Alireza Khabbazi:** project administration, supervision.

## Funding

We are thankful to the Connective Tissue Diseases Research Center of Tabriz University of Medical Sciences, Tabriz, Iran for financial support (69499).

## Ethics Statement

The study was conducted in accordance with the Declaration of Helsinki. The written permissions were obtained from the local ethics committee of Tabriz University of Medical Sciences, Tabriz, Iran (Ethic code: IR.TBZMED.REC. REC.1400.1210). All participants signed an informed consent form.

## Consent

A written informed consent was obtained from the patients and guardians to publish this report in accordance with the journal's patient consent policy.

## Conflicts of Interest

The authors declare no conflicts of interest.

## Data Availability

The data used in this study are available from the corresponding author on request.
